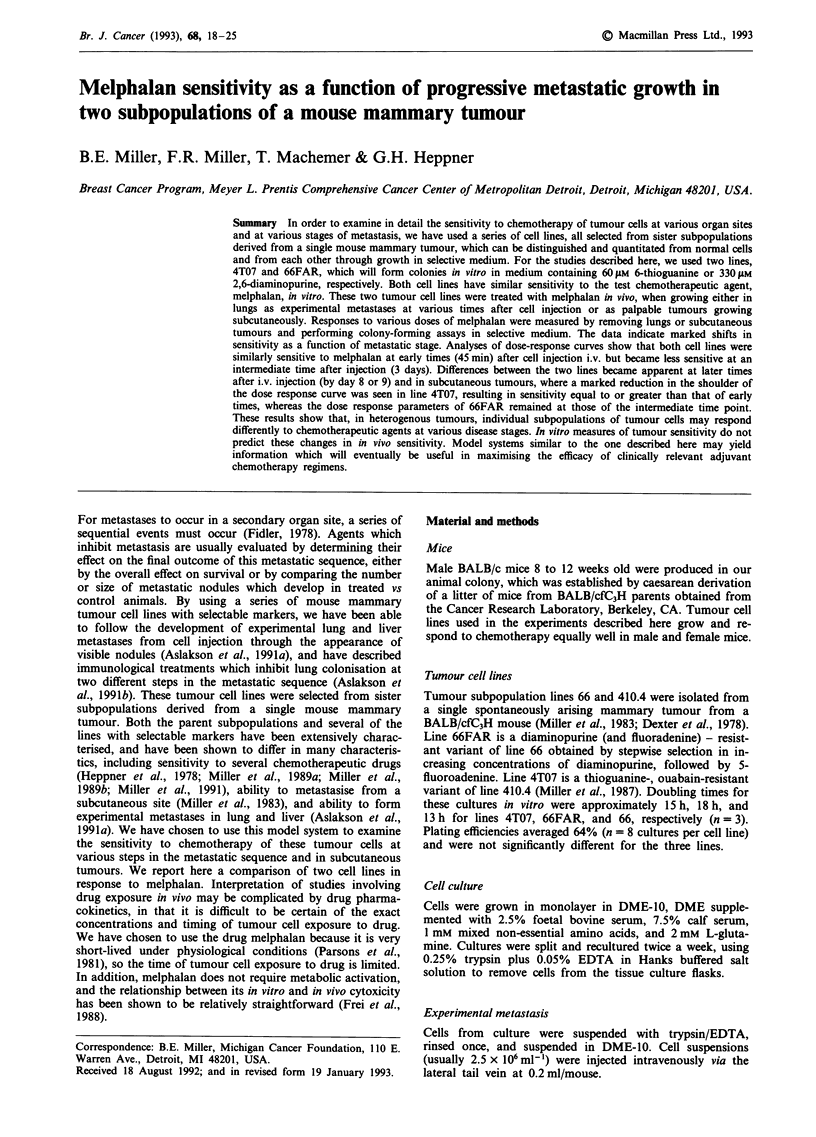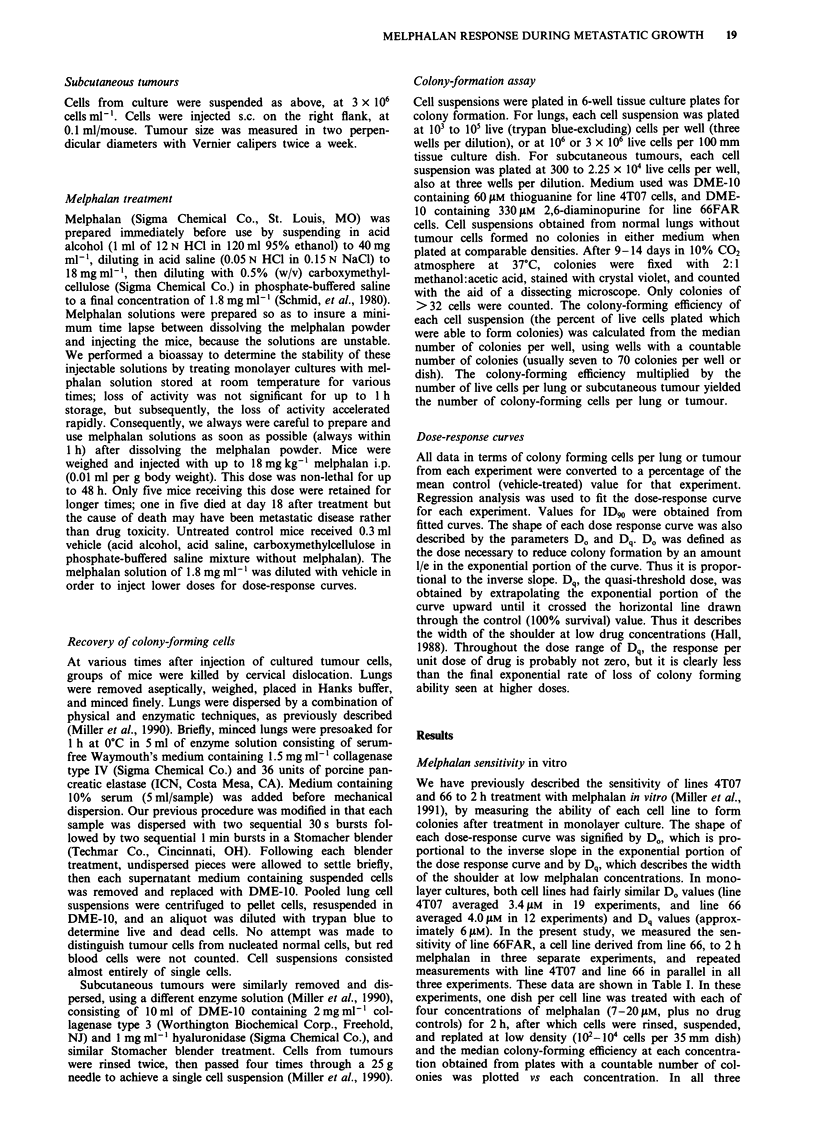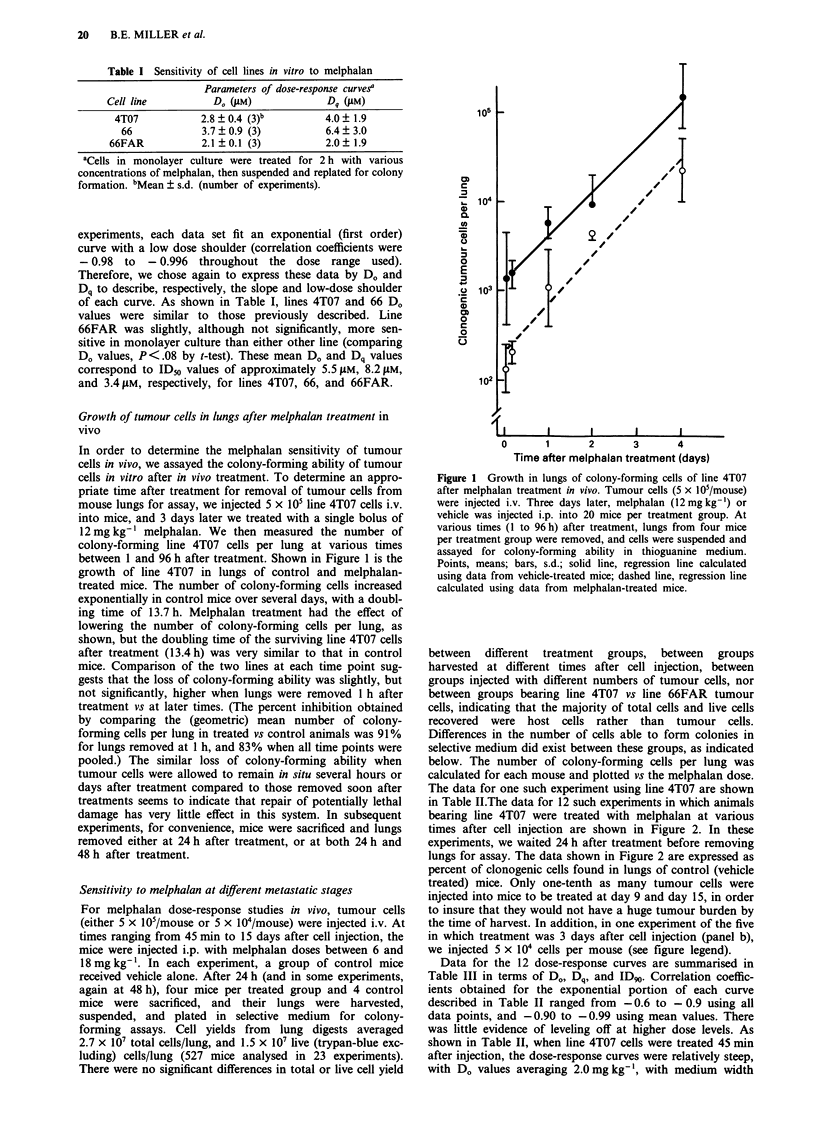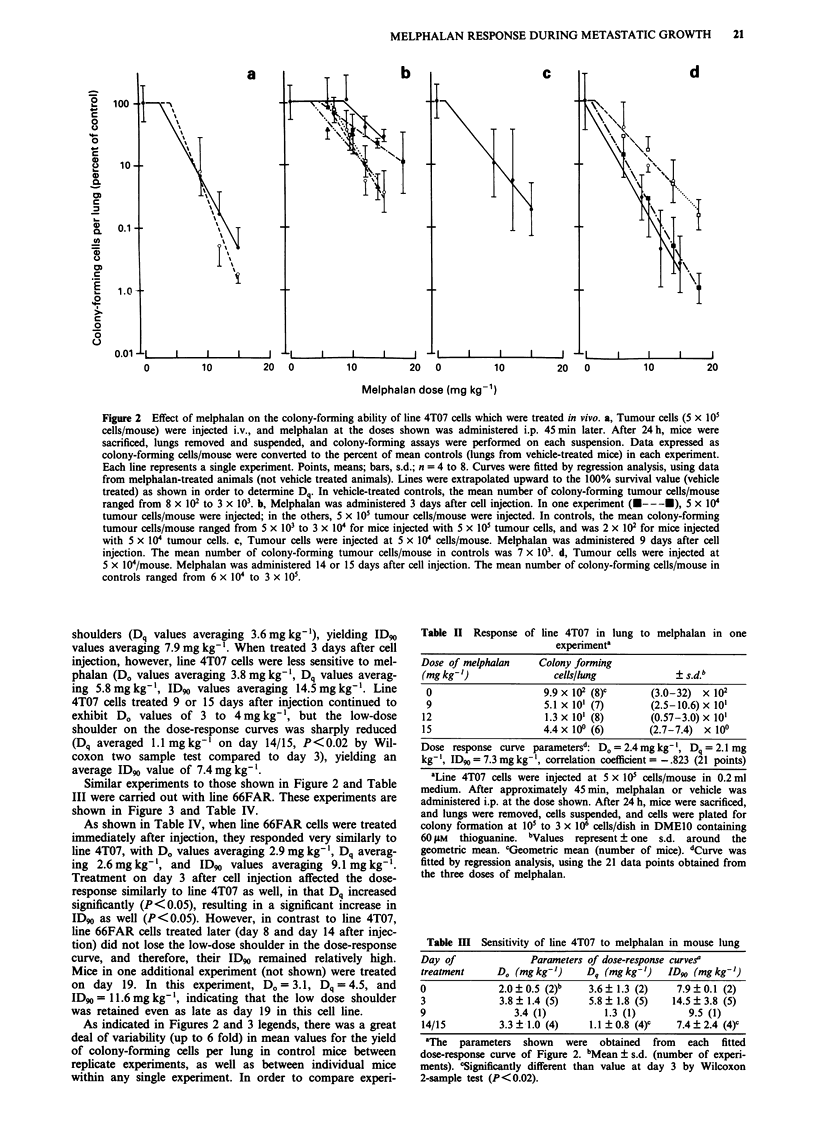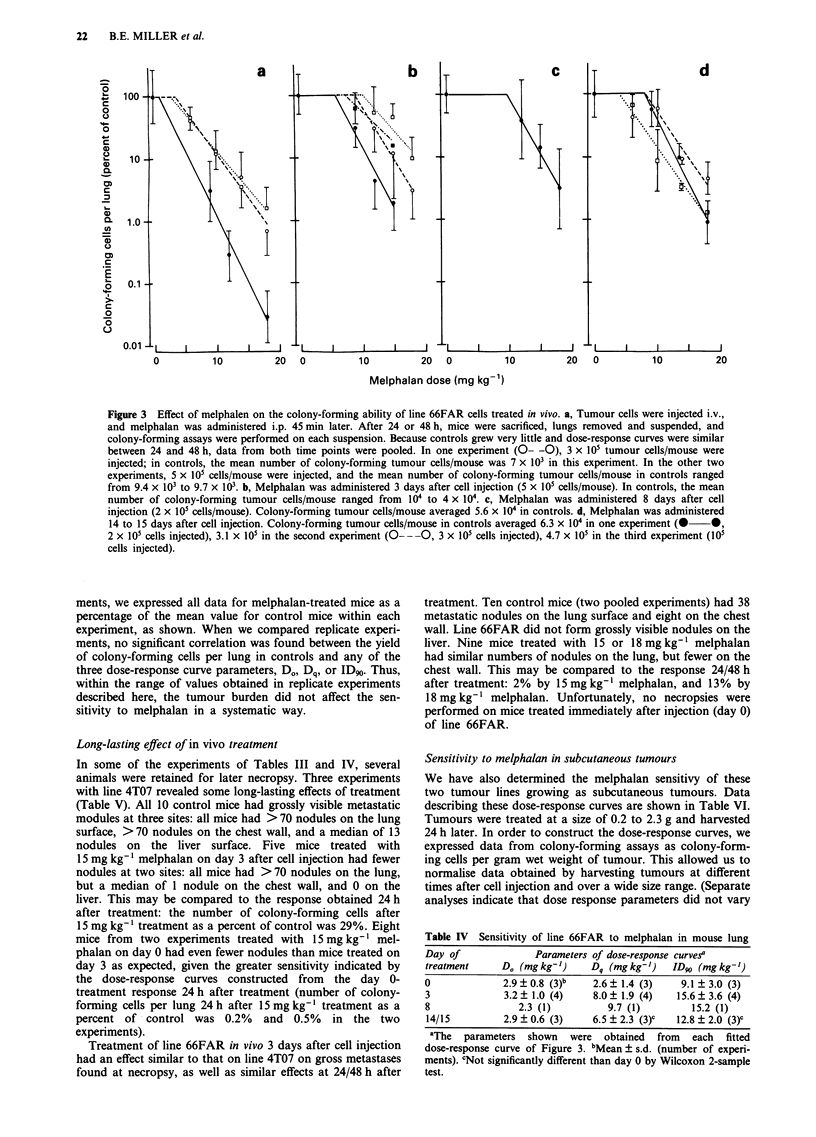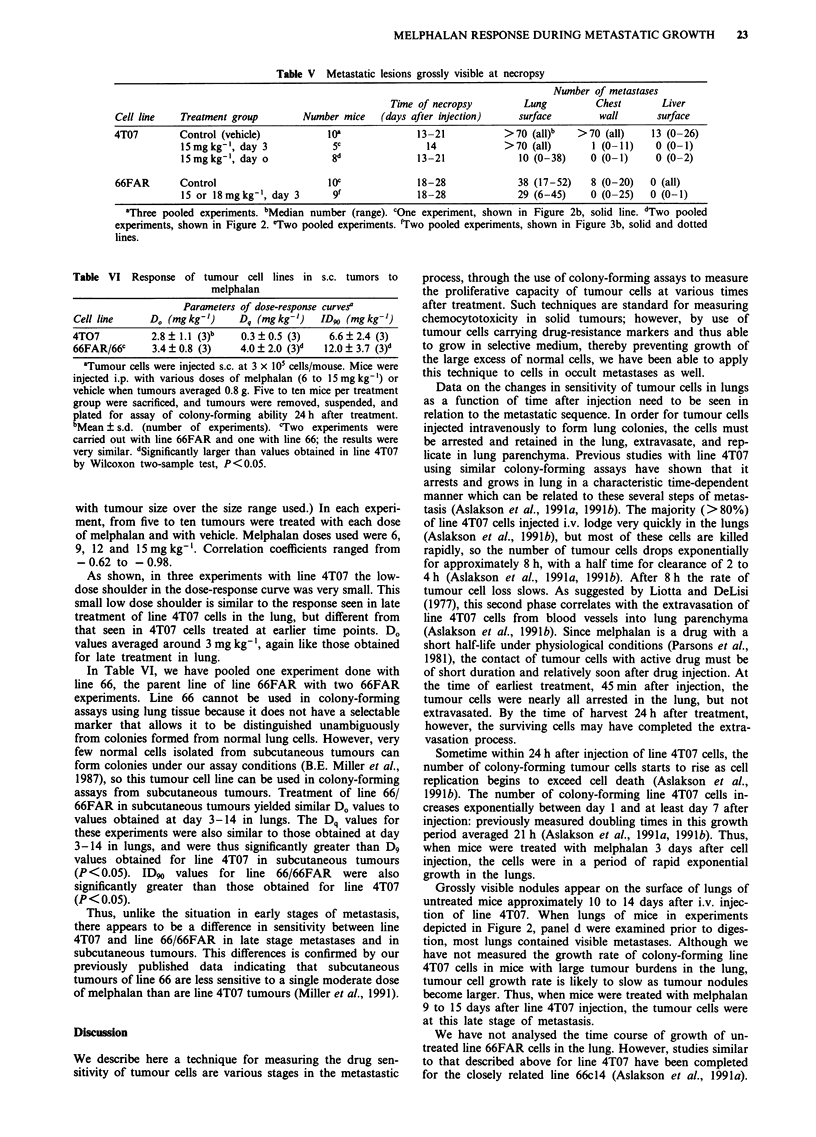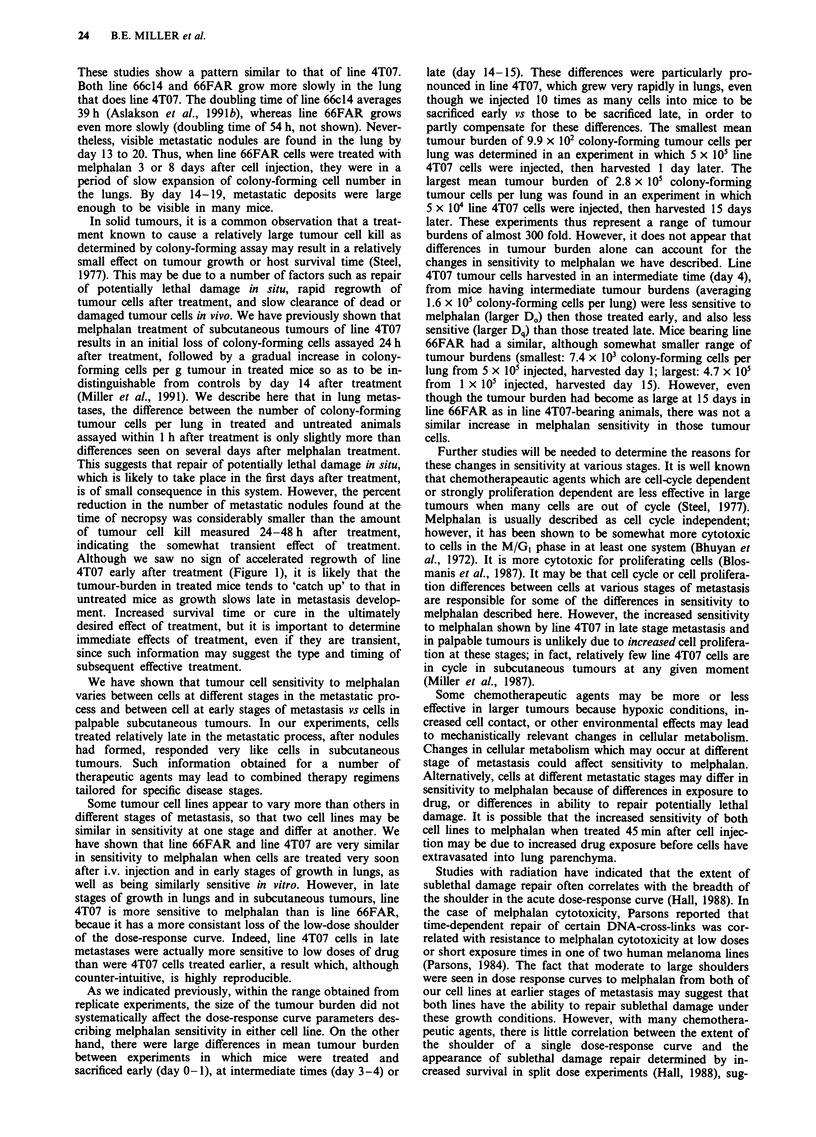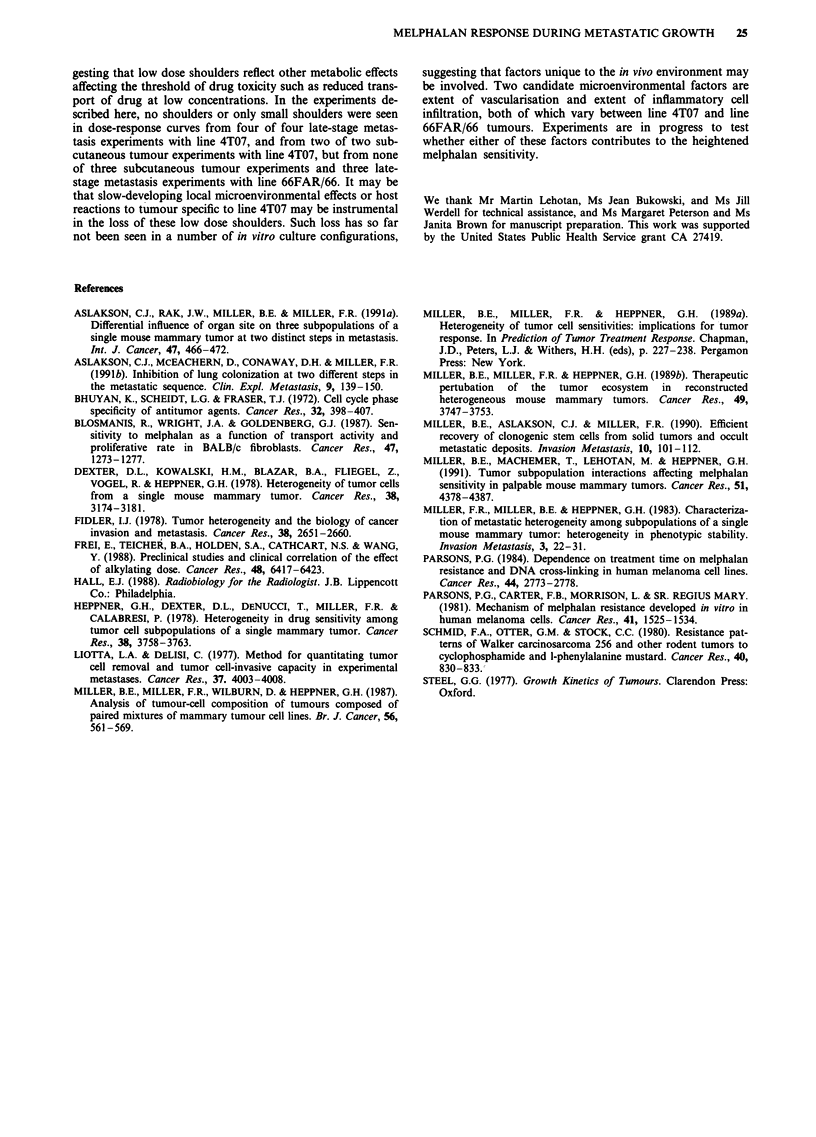# Melphalan sensitivity as a function of progressive metastatic growth in two subpopulations of a mouse mammary tumour.

**DOI:** 10.1038/bjc.1993.280

**Published:** 1993-07

**Authors:** B. E. Miller, F. R. Miller, T. Machemer, G. H. Heppner

**Affiliations:** Breast Cancer Program, Meyer L. Prentis Comprehensive Cancer Center of Metropolitan Detroit, Michigan 48201.

## Abstract

In order to examine in detail the sensitivity to chemotherapy of tumour cells at various organ sites and at various stages of metastasis, we have used a series of cell lines, all selected from sister subpopulations derived from a single mouse mammary tumour, which can be distinguished and quantitated from normal cells and from each other through growth in selective medium. For the studies described here, we used two lines, 4T07 and 66FAR, which will form colonies in vitro in medium containing 60 microM 6-thioguanine or 330 microM 2,6-diaminopurine, respectively. Both cell lines have similar sensitivity to the test chemotherapeutic agent, melphalan, in vitro. These two tumour cell lines were treated with melphalan in vivo, when growing either in lungs as experimental metastases at various times after cell injection or as palpable tumours growing subcutaneously. Responses to various doses of melphalan were measured by removing lungs or subcutaneous tumours and performing colony-forming assays in selective medium. The data indicate marked shifts in sensitivity as a function of metastatic stage. Analyses of dose-response curves show that both cell lines were similarly sensitive to melphalan at early times (45 min) after cell injection i.v. but became less sensitive at an intermediate time after injection (3 days). Differences between the two lines became apparent at later times after i.v. injection (by day 8 or 9) and in subcutaneous tumours, where a marked reduction in the shoulder of the dose response curve was seen in line 4T07, resulting in sensitivity equal to or greater than the of early times, whereas the dose response parameters of 66FAR remained at those of the intermediate time point. These results show that, in heterogeneous tumours, individual subpopulations of tumour cells may respond differently to chemotherapeutic agents at various disease stages. In vitro measures of tumour sensitivity do not predict these changes in in vivo sensitivity. Model systems similar to the one described here may yield information which will eventually be useful in maximising the efficacy of clinically relevant adjuvant chemotherapy regimens.


*Br. J. Cancer* (1993), **68**, 18–25 © Macmillan Press Ltd., 1993

### Melphalan sensitivity as a function of progressive metastatic growth in two subpopulations of a mouse mammary tumour

B.E. Miller, F.R. Miller, T. Machemer & G.H. Heppner

*Breast Cancer Program, Meyer L. Prentis Comprehensive Cancer Center of Metropolitan Detroit, Detroit, Michigan 48201, USA.*

**Summary** In order to examine in detail the sensitivity to chemotherapy of tumour cells at various organ sites and at various stages of metastasis, we have used a series of cell lines, all selected from sister subpopulations derived from a single mouse mammary tumour, which can be distinguished and quantitated from normal cells and from each other through growth in selective medium. For the studies described here, we used two lines, 4T07 and 66FAR, which will form colonies *in vitro* in medium containing 60 μM 6-thioguanine or 330 μM 2,6-diaminopurine, respectively. Both cell lines have similar sensitivity to the test chemotherapeutic agent, melphalan, *in vitro*. These two tumour cell lines were treated with melphalan *in vivo*, when growing either in lungs as experimental metastases at various times after cell injection or as palpable tumours growing subcutaneously. Responses to various doses of melphalan were measured by removing lungs or subcutaneous tumours and performing colony-forming assays in selective medium. The data indicate marked shifts in sensitivity as a function of metastatic stage. Analyses of dose-response curves show that both cell lines were similarly sensitive to melphalan at early times (45 min) after cell injection i.v. but became less sensitive at an intermediate time after injection (3 days). Differences between the two lines became apparent at later times after i.v. injection (by day 8 or 9) and in subcutaneous tumours, where a marked reduction in the shoulder of the dose response curve was seen in line 4T07, resulting in sensitivity equal to or greater than that of early times, whereas the dose response parameters of 66FAR remained at those of the intermediate time point. These results show that, in heterogenous tumours, individual subpopulations of tumour cells may respond differently to chemotherapeutic agents at various disease stages. *In vitro* measures of tumour sensitivity do not predict these changes in *in vivo* sensitivity. Model systems similar to the one described here may yield information which will eventually be useful in maximising the efficacy of clinically relevant adjuvant chemotherapy regimens.

For metastases to occur in a secondary organ site, a series of sequential events must occur (Fidler, 1978). Agents which inhibit metastasis are usually evaluated by determining their effect on the final outcome of this metastatic sequence, either by the overall effect on survival or by comparing the number or size of metastatic nodules which develop in treated *vs* control animals. By using a series of mouse mammary tumour cell lines with selectable markers, we have been able to follow the development of experimental lung and liver metastases from cell injection through the appearance of visible nodules (Aslakson *et al.*, 1991*a*), and have described immunological treatments which inhibit lung colonisation at two different steps in the metastatic sequence (Aslakson *et al.*, 1991*b*). These tumour cell lines were selected from sister subpopulations derived from a single mouse mammary tumour. Both the parent subpopulations and several of the lines with selectable markers have been extensively characterised, and have been shown to differ in many characteristics, including sensitivity to several chemotherapeutic drugs (Heppner *et al.*, 1978; Miller *et al.*, 1989*a*; Miller *et al.*, 1989*b*; Miller *et al.*, 1991), ability to metastasise from a subcutaneous site (Miller *et al.*, 1983), and ability to form experimental metastases in lung and liver (Aslakson *et al.*, 1991*a*). We have chosen to use this model system to examine the sensitivity to chemotherapy of these tumour cells at various steps in the metastatic sequence and in subcutaneous tumours. We report here a comparison of two cell lines in response to melphalan. Interpretation of studies involving drug exposure *in vivo* may be complicated by drug pharmacokinetics, in that it is difficult to be certain of the exact concentrations and timing of tumour cell exposure to drug. We have chosen to use the drug melphalan because it is very short-lived under physiological conditions (Parsons *et al.*, 1981), so the time of tumour cell exposure to drug is limited. In addition, melphalan does not require metabolic activation, and the relationship between its *in vitro* and *in vivo* cytoxicity has been shown to be relatively straightforward (Frei *et al.*, 1988).

Correspondence: B.E. Miller, Michigan Cancer Foundation, 110 E. Warren Ave., Detroit, MI 48201, USA.
Received 18 August 1992; and in revised form 19 January 1993.

### Material and methods

### *Mice*

Male BALB/c mice 8 to 12 weeks old were produced in our animal colony, which was established by caesarean derivation of a litter of mice from BALB/cf$C_3$H parents obtained from the Cancer Research Laboratory, Berkeley, CA. Tumour cell lines used in the experiments described here grow and respond to chemotherapy equally well in male and female mice.

### *Tumour cell lines*

Tumour subpopulation lines 66 and 410.4 were isolated from a single spontaneously arising mammary tumour from a BALB/cf$C_3$H mouse (Miller *et al.*, 1983; Dexter *et al.*, 1978). Line 66FAR is a diaminopurine (and fluoradenine) – resistant variant of line 66 obtained by stepwise selection in increasing concentrations of diaminopurine, followed by 5-fluoroadenine. Line 4T07 is a thioguanine-, ouabain-resistant variant of line 410.4 (Miller *et al.*, 1987). Doubling times for these cultures *in vitro* were approximately 15 h, 18 h, and 13 h for lines 4T07, 66FAR, and 66, respectively ($n = 3$). Plating efficiencies averaged 64% ($n = 8$ cultures per cell line) and were not significantly different for the three lines.

### *Cell culture*

Cells were grown in monolayer in DME-10, DME supplemented with 2.5% foetal bovine serum, 7.5% calf serum, 1 mM mixed non-essential amino acids, and 2 mM L-glutamine. Cultures were split and recultured twice a week, using 0.25% trypsin plus 0.05% EDTA in Hanks buffered salt solution to remove cells from the tissue culture flasks.

### *Experimental metastasis*

Cells from culture were suspended with trypsin/EDTA, rinsed once, and suspended in DME-10. Cell suspensions (usually $2.5 \times 10^6\ ml^{-1}$) were injected intravenously *via* the lateral tail vein at 0.2 ml/mouse.

*Subcutaneous tumours*

Cells from culture were suspended as above, at $3 \times 10^6$ cells $ml^{-1}$. Cells were injected s.c. on the right flank, at 0.1 ml/mouse. Tumour size was measured in two perpendicular diameters with Vernier calipers twice a week.

*Melphalan treatment*

Melphalan (Sigma Chemical Co., St. Louis, MO) was prepared immediately before use by suspending in acid alcohol (1 ml of 12 N HCl in 120 ml 95% ethanol) to 40 mg $ml^{-1}$, diluting in acid saline (0.05 N HCl in 0.15 N NaCl) to 18 mg $ml^{-1}$, then diluting with 0.5% (w/v) carboxymethylcellulose (Sigma Chemical Co.) in phosphate-buffered saline to a final concentration of 1.8 mg $ml^{-1}$ (Schmid, *et al.*, 1980). Melphalan solutions were prepared so as to insure a minimum time lapse between dissolving the melphalan powder and injecting the mice, because the solutions are unstable. We performed a bioassay to determine the stability of these injectable solutions by treating monolayer cultures with melphalan solution stored at room temperature for various times; loss of activity was not significant for up to 1 h storage, but subsequently, the loss of activity accelerated rapidly. Consequently, we always were careful to prepare and use melphalan solutions as soon as possible (always within 1 h) after dissolving the melphalan powder. Mice were weighed and injected with up to 18 mg $kg^{-1}$ melphalan i.p. (0.01 ml per g body weight). This dose was non-lethal for up to 48 h. Only five mice receiving this dose were retained for longer times; one in five died at day 18 after treatment but the cause of death may have been metastatic disease rather than drug toxicity. Untreated control mice received 0.3 ml vehicle (acid alcohol, acid saline, carboxymethylcellulose in phosphate-buffered saline mixture without melphalan). The melphalan solution of 1.8 mg $ml^{-1}$ was diluted with vehicle in order to inject lower doses for dose-response curves.

*Recovery of colony-forming cells*

At various times after injection of cultured tumour cells, groups of mice were killed by cervical dislocation. Lungs were removed aseptically, weighed, placed in Hanks buffer, and minced finely. Lungs were dispersed by a combination of physical and enzymatic techniques, as previously described (Miller *et al.*, 1990). Briefly, minced lungs were presoaked for 1 h at 0°C in 5 ml of enzyme solution consisting of serum-free Waymouth's medium containing 1.5 mg $ml^{-1}$ collagenase type IV (Sigma Chemical Co.) and 36 units of porcine pancreatic elastase (ICN, Costa Mesa, CA). Medium containing 10% serum (5 ml/sample) was added before mechanical dispersion. Our previous procedure was modified in that each sample was dispersed with two sequential 30 s bursts followed by two sequential 1 min bursts in a Stomacher blender (Techmar Co., Cincinnati, OH). Following each blender treatment, undispersed pieces were allowed to settle briefly, then each supernatant medium containing suspended cells was removed and replaced with DME-10. Pooled lung cell suspensions were centrifuged to pellet cells, resuspended in DME-10, and an aliquot was diluted with trypan blue to determine live and dead cells. No attempt was made to distinguish tumour cells from nucleated normal cells, but red blood cells were not counted. Cell suspensions consisted almost entirely of single cells.

Subcutaneous tumours were similarly removed and dispersed, using a different enzyme solution (Miller *et al.*, 1990), consisting of 10 ml of DME-10 containing 2 mg $ml^{-1}$ collagenase type 3 (Worthington Biochemical Corp., Freehold, NJ) and 1 mg $ml^{-1}$ hyaluronidase (Sigma Chemical Co.), and similar Stomacher blender treatment. Cells from tumours were rinsed twice, then passed four times through a 25 g needle to achieve a single cell suspension (Miller *et al.*, 1990).

*Colony-formation assay*

Cell suspensions were plated in 6-well tissue culture plates for colony formation. For lungs, each cell suspension was plated at $10^3$ to $10^5$ live (trypan blue-excluding) cells per well (three wells per dilution), or at $10^6$ or $3 \times 10^6$ live cells per 100 mm tissue culture dish. For subcutaneous tumours, each cell suspension was plated at 300 to $2.25 \times 10^4$ live cells per well, also at three wells per dilution. Medium used was DME-10 containing 60 $\mu$M thioguanine for line 4T07 cells, and DME-10 containing 330 $\mu$M 2,6-diaminopurine for line 66FAR cells. Cell suspensions obtained from normal lungs without tumour cells formed no colonies in either medium when plated at comparable densities. After 9–14 days in 10% $CO_2$ atmosphere at 37°C, colonies were fixed with 2:1 methanol:acetic acid, stained with crystal violet, and counted with the aid of a dissecting microscope. Only colonies of $>32$ cells were counted. The colony-forming efficiency of each cell suspension (the percent of live cells plated which were able to form colonies) was calculated from the median number of colonies per well, using wells with a countable number of colonies (usually seven to 70 colonies per well or dish). The colony-forming efficiency multiplied by the number of live cells per lung or subcutaneous tumour yielded the number of colony-forming cells per lung or tumour.

*Dose-response curves*

All data in terms of colony forming cells per lung or tumour from each experiment were converted to a percentage of the mean control (vehicle-treated) value for that experiment. Regression analysis was used to fit the dose-response curve for each experiment. Values for $ID_{90}$ were obtained from fitted curves. The shape of each dose response curve was also described by the parameters $D_o$ and $D_q$. $D_o$ was defined as the dose necessary to reduce colony formation by an amount 1/e in the exponential portion of the curve. Thus it is proportional to the inverse slope. $D_q$, the quasi-threshold dose, was obtained by extrapolating the exponential portion of the curve upward until it crossed the horizontal line drawn through the control (100% survival) value. Thus it describes the width of the shoulder at low drug concentrations (Hall, 1988). Throughout the dose range of $D_q$, the response per unit dose of drug is probably not zero, but it is clearly less than the final exponential rate of loss of colony forming ability seen at higher doses.

### Results

*Melphalan sensitivity* in vitro

We have previously described the sensitivity of lines 4T07 and 66 to 2 h treatment with melphalan *in vitro* (Miller *et al.*, 1991), by measuring the ability of each cell line to form colonies after treatment in monolayer culture. The shape of each dose-response curve was signified by $D_o$, which is proportional to the inverse slope in the exponential portion of the dose response curve and by $D_q$, which describes the width of the shoulder at low melphalan concentrations. In monolayer cultures, both cell lines had fairly similar $D_o$ values (line 4T07 averaged 3.4 $\mu$M in 19 experiments, and line 66 averaged 4.0 $\mu$M in 12 experiments) and $D_q$ values (approximately 6 $\mu$M). In the present study, we measured the sensitivity of line 66FAR, a cell line derived from line 66, to 2 h melphalan in three separate experiments, and repeated measurements with line 4T07 and line 66 in parallel in all three experiments. These data are shown in Table I. In these experiments, one dish per cell line was treated with each of four concentrations of melphalan (7–20 $\mu$M, plus no drug controls) for 2 h, after which cells were rinsed, suspended, and replated at low density ($10^2$–$10^4$ cells per 35 mm dish) and the median colony-forming efficiency at each concentration obtained from plates with a countable number of colonies was plotted *vs* each concentration. In all three

**Table I** Sensitivity of cell lines *in vitro* to melphalan

| Cell line | Parameters of dose-response curves[a] $D_o$ (μM) | $D_q$ (μM) |
|---|---|---|
| 4T07 | 2.8 ± 0.4 (3)[b] | 4.0 ± 1.9 |
| 66 | 3.7 ± 0.9 (3) | 6.4 ± 3.0 |
| 66FAR | 2.1 ± 0.1 (3) | 2.0 ± 1.9 |

[a]Cells in monolayer culture were treated for 2 h with various concentrations of melphalan, then suspended and replated for colony formation. [b]Mean ± s.d. (number of experiments).

experiments, each data set fit an exponential (first order) curve with a low dose shoulder (correlation coefficients were −0.98 to −0.996 throughout the dose range used). Therefore, we chose again to express these data by $D_o$ and $D_q$ to describe, respectively, the slope and low-dose shoulder of each curve. As shown in Table I, lines 4T07 and 66 $D_o$ values were similar to those previously described. Line 66FAR was slightly, although not significantly, more sensitive in monolayer culture than either other line (comparing $D_o$ values, $P<.08$ by *t*-test). These mean $D_o$ and $D_q$ values correspond to $ID_{50}$ values of approximately 5.5 μM, 8.2 μM, and 3.4 μM, respectively, for lines 4T07, 66, and 66FAR.

*Growth of tumour cells in lungs after melphalan treatment* in vivo

In order to determine the melphalan sensitivity of tumour cells *in vivo*, we assayed the colony-forming ability of tumour cells *in vitro* after *in vivo* treatment. To determine an appropriate time after treatment for removal of tumour cells from mouse lungs for assay, we injected $5 \times 10^5$ line 4T07 cells i.v. into mice, and 3 days later we treated with a single bolus of 12 mg $kg^{-1}$ melphalan. We then measured the number of colony-forming line 4T07 cells per lung at various times between 1 and 96 h after treatment. Shown in Figure 1 is the growth of line 4T07 in lungs of control and melphalan-treated mice. The number of colony-forming cells increased exponentially in control mice over several days, with a doubling time of 13.7 h. Melphalan treatment had the effect of lowering the number of colony-forming cells per lung, as shown, but the doubling time of the surviving line 4T07 cells after treatment (13.4 h) was very similar to that in control mice. Comparison of the two lines at each time point suggests that the loss of colony-forming ability was slightly, but not significantly, higher when lungs were removed 1 h after treatment *vs* at later times. (The percent inhibition obtained by comparing the (geometric) mean number of colony-forming cells per lung in treated *vs* control animals was 91% for lungs removed at 1 h, and 83% when all time points were pooled.) The similar loss of colony-forming ability when tumour cells were allowed to remain *in situ* several hours or days after treatment compared to those removed soon after treatments seems to indicate that repair of potentially lethal damage has very little effect in this system. In subsequent experiments, for convenience, mice were sacrificed and lungs removed either at 24 h after treatment, or at both 24 h and 48 h after treatment.

*Sensitivity to melphalan at different metastatic stages*

For melphalan dose-response studies *in vivo*, tumour cells (either $5 \times 10^5$/mouse or $5 \times 10^4$/mouse) were injected i.v. At times ranging from 45 min to 15 days after cell injection, the mice were injected i.p. with melphalan doses between 6 and 18 mg $kg^{-1}$. In each experiment, a group of control mice received vehicle alone. After 24 h (and in some experiments, again at 48 h), four mice per treated group and 4 control mice were sacrificed, and their lungs were harvested, suspended, and plated in selective medium for colony-forming assays. Cell yields from lung digests averaged $2.7 \times 10^7$ total cells/lung, and $1.5 \times 10^7$ live (trypan-blue excluding) cells/lung (527 mice analysed in 23 experiments). There were no significant differences in total or live cell yield

**Figure 1** Growth in lungs of colony-forming cells of line 4T07 after melphalan treatment *in vivo*. Tumour cells ($5 \times 10^5$/mouse) were injected i.v. Three days later, melphalan (12 mg $kg^{-1}$) or vehicle was injected i.p. into 20 mice per treatment group. At various times (1 to 96 h) after treatment, lungs from four mice per treatment group were removed, and cells were suspended and assayed for colony-forming ability in thioguanine medium. Points, means; bars, s.d.; solid line, regression line calculated using data from vehicle-treated mice; dashed line, regression line calculated using data from melphalan-treated mice.

between different treatment groups, between groups harvested at different times after cell injection, between groups injected with different numbers of tumour cells, nor between groups bearing line 4T07 *vs* line 66FAR tumour cells, indicating that the majority of total cells and live cells recovered were host cells rather than tumour cells. Differences in the number of cells able to form colonies in selective medium did exist between these groups, as indicated below. The number of colony-forming cells per lung was calculated for each mouse and plotted *vs* the melphalan dose. The data for one such experiment using line 4T07 are shown in Table II.The data for 12 such experiments in which animals bearing line 4T07 were treated with melphalan at various times after cell injection are shown in Figure 2. In these experiments, we waited 24 h after treatment before removing lungs for assay. The data shown in Figure 2 are expressed as percent of clonogenic cells found in lungs of control (vehicle treated) mice. Only one-tenth as many tumour cells were injected into mice to be treated at day 9 and day 15, in order to insure that they would not have a huge tumour burden by the time of harvest. In addition, in one experiment of the five in which treatment was 3 days after cell injection (panel b), we injected $5 \times 10^4$ cells per mouse (see figure legend).

Data for the 12 dose-response curves are summarised in Table III in terms of $D_o$, $D_q$, and $ID_{90}$. Correlation coefficients obtained for the exponential portion of each curve described in Table II ranged from −0.6 to −0.9 using all data points, and −0.90 to −0.99 using mean values. There was little evidence of leveling off at higher dose levels. As shown in Table II, when line 4T07 cells were treated 45 min after injection, the dose-response curves were relatively steep, with $D_o$ values averaging 2.0 mg $kg^{-1}$, with medium width

**Figure 2** Effect of melphalan on the colony-forming ability of line 4T07 cells which were treated *in vivo*. **a**, Tumour cells ($5 \times 10^5$ cells/mouse) were injected i.v., and melphalan at the doses shown was administered i.p. 45 min later. After 24 h, mice were sacrificed, lungs removed and suspended, and colony-forming assays were performed on each suspension. Data expressed as colony-forming cells/mouse were converted to the percent of mean controls (lungs from vehicle-treated mice) in each experiment. Each line represents a single experiment. Points, means; bars, s.d.; $n = 4$ to 8. Curves were fitted by regression analysis, using data from melphalan-treated animals (not vehicle treated animals). Lines were extrapolated upward to the 100% survival value (vehicle treated) as shown in order to determine $D_q$. In vehicle-treated controls, the mean number of colony-forming tumour cells/mouse ranged from $8 \times 10^2$ to $3 \times 10^3$. **b**, Melphalan was administered 3 days after cell injection. In one experiment (■---■), $5 \times 10^4$ tumour cells/mouse were injected; in the others, $5 \times 10^5$ tumour cells/mouse were injected. In controls, the mean colony-forming tumour cells/mouse ranged from $5 \times 10^3$ to $3 \times 10^4$ for mice injected with $5 \times 10^5$ tumour cells, and was $2 \times 10^2$ for mice injected with $5 \times 10^4$ tumour cells. **c**, Tumour cells were injected at $5 \times 10^4$ cells/mouse. Melphalan was administered 9 days after cell injection. The mean number of colony-forming tumour cells/mouse in controls was $7 \times 10^3$. **d**, Tumour cells were injected at $5 \times 10^4$/mouse. Melphalan was administered 14 or 15 days after cell injection. The mean number of colony-forming cells/mouse in controls ranged from $6 \times 10^4$ to $3 \times 10^5$.

shoulders ($D_q$ values averaging 3.6 mg kg$^{-1}$), yielding $ID_{90}$ values averaging 7.9 mg kg$^{-1}$. When treated 3 days after cell injection, however, line 4T07 cells were less sensitive to melphalan ($D_o$ values averaging 3.8 mg kg$^{-1}$, $D_q$ values averaging 5.8 mg kg$^{-1}$, $ID_{90}$ values averaging 14.5 mg kg$^{-1}$. Line 4T07 cells treated 9 or 15 days after injection continued to exhibit $D_o$ values of 3 to 4 mg kg$^{-1}$, but the low-dose shoulder on the dose-response curves was sharply reduced ($D_q$ averaged 1.1 mg kg$^{-1}$ on day 14/15, $P < 0.02$ by Wilcoxon two sample test compared to day 3), yielding an average $ID_{90}$ value of 7.4 mg kg$^{-1}$.

Similar experiments to those shown in Figure 2 and Table III were carried out with line 66FAR. These experiments are shown in Figure 3 and Table IV.

As shown in Table IV, when line 66FAR cells were treated immediately after injection, they responded very similarly to line 4T07, with $D_o$ values averaging 2.9 mg kg$^{-1}$, $D_q$ averaging 2.6 mg kg$^{-1}$, and $ID_{90}$ values averaging 9.1 mg kg$^{-1}$. Treatment on day 3 after cell injection affected the dose-response similarly to line 4T07 as well, in that $D_q$ increased significantly ($P < 0.05$), resulting in a significant increase in $ID_{90}$ as well ($P < 0.05$). However, in contrast to line 4T07, line 66FAR cells treated later (day 8 and day 14 after injection) did not lose the low-dose shoulder in the dose-response curve, and therefore, their $ID_{90}$ remained relatively high. Mice in one additional experiment (not shown) were treated on day 19. In this experiment, $D_o = 3.1$, $D_q = 4.5$, and $ID_{90} = 11.6$ mg kg$^{-1}$, indicating that the low dose shoulder was retained even as late as day 19 in this cell line.

As indicated in Figures 2 and 3 legends, there was a great deal of variability (up to 6 fold) in mean values for the yield of colony-forming cells per lung in control mice between replicate experiments, as well as between individual mice within any single experiment. In order to compare experi-

**Table II** Response of line 4T07 in lung to melphalan in one experiment[a]

| *Dose of melphalan (mg kg⁻¹)* | *Colony forming cells/lung* | *± s.d.*[b] |
|---|---|---|
| 0 | $9.9 \times 10^2$ (8)[c] | (3.0–32) $\times 10^2$ |
| 9 | $5.1 \times 10^1$ (7) | (2.5–10.6) $\times 10^1$ |
| 12 | $1.3 \times 10^1$ (8) | (0.57–3.0) $\times 10^1$ |
| 15 | $4.4 \times 10^0$ (6) | (2.7–7.4) $\times 10^0$ |

Dose response curve parameters[d]: $D_o = 2.4$ mg kg$^{-1}$, $D_q = 2.1$ mg kg$^{-1}$, $ID_{90} = 7.3$ mg kg$^{-1}$, correlation coefficient = −.823 (21 points)

[a]Line 4T07 cells were injected at $5 \times 10^5$ cells/mouse in 0.2 ml medium. After approximately 45 min, melphalan or vehicle was administered i.p. at the dose shown. After 24 h, mice were sacrificed, and lungs were removed, cells suspended, and cells were plated for colony formation at $10^5$ to $3 \times 10^6$ cells/dish in DME10 containing 60 μM thioguanine. [b]Values represent ± one s.d. around the geometric mean. [c]Geometric mean (number of mice). [d]Curve was fitted by regression analysis, using the 21 data points obtained from the three doses of melphalan.

**Table III** Sensitivity of line 4T07 to melphalan in mouse lung

| *Day of treatment* | *Parameters of dose-response curves*[a] *$D_o$ (mg kg⁻¹)* | *$D_q$ (mg kg⁻¹)* | *$ID_{90}$ (mg kg⁻¹)* |
|---|---|---|---|
| 0 | 2.0 ± 0.5 (2)[b] | 3.6 ± 1.3 (2) | 7.9 ± 0.1 (2) |
| 3 | 3.8 ± 1.4 (5) | 5.8 ± 1.8 (5) | 14.5 ± 3.8 (5) |
| 9 | 3.4 (1) | 1.3 (1) | 9.5 (1) |
| 14/15 | 3.3 ± 1.0 (4) | 1.1 ± 0.8 (4)[c] | 7.4 ± 2.4 (4)[c] |

[a]The parameters shown were obtained from each fitted dose-response curve of Figure 2. [b]Mean ± s.d. (number of experiments). [c]Significantly different than value at day 3 by Wilcoxon 2-sample test ($P < 0.02$).

**Figure 3** Effect of melphalen on the colony-forming ability of line 66FAR cells treated *in vivo*. **a**, Tumour cells were injected i.v., and melphalan was administered i.p. 45 min later. After 24 or 48 h, mice were sacrificed, lungs removed and suspended, and colony-forming assays were performed on each suspension. Because controls grew very little and dose-response curves were similar between 24 and 48 h, data from both time points were pooled. In one experiment (O- -O), $3 \times 10^5$ tumour cells/mouse were injected; in controls, the mean number of colony-forming tumour cells/mouse was $7 \times 10^3$ in this experiment. In the other two experiments, $5 \times 10^5$ cells/mouse were injected, and the mean number of colony-forming tumour cells/mouse in controls ranged from $9.4 \times 10^3$ to $9.7 \times 10^3$. **b**, Melphalan was administered 3 days after cell injection ($5 \times 10^5$ cells/mouse). In controls, the mean number of colony-forming tumour cells/mouse ranged from $10^4$ to $4 \times 10^4$. **c**, Melphalan was administered 8 days after cell injection ($2 \times 10^5$ cells/mouse). Colony-forming tumour cells/mouse averaged $5.6 \times 10^4$ in controls. **d**, Melphalan was administered 14 to 15 days after cell injection. Colony-forming tumour cells/mouse in controls averaged $6.3 \times 10^4$ in one experiment (●——●, $2 \times 10^5$ cells injected), $3.1 \times 10^5$ in the second experiment (O---O, $3 \times 10^5$ cells injected), $4.7 \times 10^5$ in the third experiment ($10^5$ cells injected).

ments, we expressed all data for melphalan-treated mice as a percentage of the mean value for control mice within each experiment, as shown. When we compared replicate experiments, no significant correlation was found between the yield of colony-forming cells per lung in controls and any of the three dose-response curve parameters, $D_o$, $D_q$, or $ID_{90}$. Thus, within the range of values obtained in replicate experiments described here, the tumour burden did not affect the sensitivity to melphalan in a systematic way.

### *Long-lasting effect of* in vivo *treatment*

In some of the experiments of Tables III and IV, several animals were retained for later necropsy. Three experiments with line 4T07 revealed some long-lasting effects of treatment (Table V). All 10 control mice had grossly visible metastatic modules at three sites: all mice had $>70$ nodules on the lung surface, $>70$ nodules on the chest wall, and a median of 13 nodules on the liver surface. Five mice treated with 15 mg kg$^{-1}$ melphalan on day 3 after cell injection had fewer nodules at two sites: all mice had $>70$ nodules on the lung, but a median of 1 nodule on the chest wall, and 0 on the liver. This may be compared to the response obtained 24 h after treatment: the number of colony-forming cells after 15 mg kg$^{-1}$ treatment as a percent of control was 29%. Eight mice from two experiments treated with 15 mg kg$^{-1}$ melphalan on day 0 had even fewer nodules than mice treated on day 3 as expected, given the greater sensitivity indicated by the dose-response curves constructed from the day 0-treatment response 24 h after treatment (number of colony-forming cells per lung 24 h after 15 mg kg$^{-1}$ treatment as a percent of control was 0.2% and 0.5% in the two experiments).

Treatment of line 66FAR *in vivo* 3 days after cell injection had an effect similar to that on line 4T07 on gross metastases found at necropsy, as well as similar effects at 24/48 h after treatment. Ten control mice (two pooled experiments) had 38 metastatic nodules on the lung surface and eight on the chest wall. Line 66FAR did not form grossly visible nodules on the liver. Nine mice treated with 15 or 18 mg kg$^{-1}$ melphalan had similar numbers of nodules on the lung, but fewer on the chest wall. This may be compared to the response 24/48 h after treatment: 2% by 15 mg kg$^{-1}$ melphalan, and 13% by 18 mg kg$^{-1}$ melphalan. Unfortunately, no necropsies were performed on mice treated immediately after injection (day 0) of line 66FAR.

### *Sensitivity to melphalan in subcutaneous tumours*

We have also determined the melphalan sensitivy of these two tumour lines growing as subcutaneous tumours. Data describing these dose-response curves are shown in Table VI. Tumours were treated at a size of 0.2 to 2.3 g and harvested 24 h later. In order to construct the dose-response curves, we expressed data from colony-forming assays as colony-forming cells per gram wet weight of tumour. This allowed us to normalise data obtained by harvesting tumours at different times after cell injection and over a wide size range. (Separate analyses indicate that dose response parameters did not vary

**Table IV** Sensitivity of line 66FAR to melphalan in mouse lung

| *Day of treatment* | *Parameters of dose-response curves*[a] $D_o$ *(mg kg$^{-1}$)* | $D_q$ *(mg kg$^{-1}$)* | $ID_{90}$ *(mg kg$^{-1}$)* |
|---|---|---|---|
| 0 | 2.9 ± 0.8 (3)[b] | 2.6 ± 1.4 (3) | 9.1 ± 3.0 (3) |
| 3 | 3.2 ± 1.0 (4) | 8.0 ± 1.9 (4) | 15.6 ± 3.6 (4) |
| 8 | 2.3 (1) | 9.7 (1) | 15.2 (1) |
| 14/15 | 2.9 ± 0.6 (3) | 6.5 ± 2.3 (3)[c] | 12.8 ± 2.0 (3)[c] |

[a]The parameters shown were obtained from each fitted dose-response curve of Figure 3. [b]Mean ± s.d. (number of experiments). [c]Not significantly different than day 0 by Wilcoxon 2-sample test.

**Table V** Metastatic lesions grossly visible at necropsy

| Cell line | Treatment group | Number mice | Time of necropsy (days after injection) | Number of metastases: Lung surface | Chest wall | Liver surface |
|---|---|---|---|---|---|---|
| 4T07 | Control (vehicle) | 10[a] | 13–21 | >70 (all)[b] | >70 (all) | 13 (0–26) |
| | 15 mg kg$^{-1}$, day 3 | 5[c] | 14 | >70 (all) | 1 (0–11) | 0 (0–1) |
| | 15 mg kg$^{-1}$, day o | 8[d] | 13–21 | 10 (0–38) | 0 (0–1) | 0 (0–2) |
| 66FAR | Control | 10[e] | 18–28 | 38 (17–52) | 8 (0–20) | 0 (all) |
| | 15 or 18 mg kg$^{-1}$, day 3 | 9[f] | 18–28 | 29 (6–45) | 0 (0–25) | 0 (0–1) |

[a]Three pooled experiments. [b]Median number (range). [c]One experiment, shown in Figure 2b, solid line. [d]Two pooled experiments, shown in Figure 2. [e]Two pooled experiments. [f]Two pooled experiments, shown in Figure 3b, solid and dotted lines.

**Table VI** Response of tumour cell lines in s.c. tumors to melphalan

| Cell line | Parameters of dose-response curves[a]: $D_o$ (mg kg$^{-1}$) | $D_q$ (mg kg$^{-1}$) | $ID_{90}$ (mg kg$^{-1}$) |
|---|---|---|---|
| 4TO7 | 2.8 ± 1.1 (3)[b] | 0.3 ± 0.5 (3) | 6.6 ± 2.4 (3) |
| 66FAR/66[c] | 3.4 ± 0.8 (3) | 4.0 ± 2.0 (3)[d] | 12.0 ± 3.7 (3)[d] |

[a]Tumour cells were injected s.c. at $3 \times 10^5$ cells/mouse. Mice were injected i.p. with various doses of melphalan (6 to 15 mg kg$^{-1}$) or vehicle when tumours averaged 0.8 g. Five to ten mice per treatment group were sacrificed, and tumours were removed, suspended, and plated for assay of colony-forming ability 24 h after treatment. [b]Mean ± s.d. (number of experiments). [c]Two experiments were carried out with line 66FAR and one with line 66; the results were very similar. [d]Significantly larger than values obtained in line 4T07 by Wilcoxon two-sample test, $P<0.05$.

with tumour size over the size range used.) In each experiment, from five to ten tumours were treated with each dose of melphalan and with vehicle. Melphalan doses used were 6, 9, 12 and 15 mg kg$^{-1}$. Correlation coefficients ranged from − 0.62 to − 0.98.

As shown, in three experiments with line 4T07 the low-dose shoulder in the dose-response curve was very small. This small low dose shoulder is similar to the response seen in late treatment of line 4T07 cells in the lung, but different from that seen in 4T07 cells treated at earlier time points. $D_o$ values averaged around 3 mg kg$^{-1}$, again like those obtained for late treatment in lung.

In Table VI, we have pooled one experiment done with line 66, the parent line of line 66FAR with two 66FAR experiments. Line 66 cannot be used in colony-forming assays using lung tissue because it does not have a selectable marker that allows it to be distinguished unambiguously from colonies formed from normal lung cells. However, very few normal cells isolated from subcutaneous tumours can form colonies under our assay conditions (B.E. Miller *et al.*, 1987), so this tumour cell line can be used in colony-forming assays from subcutaneous tumours. Treatment of line 66/66FAR in subcutaneous tumours yielded similar $D_o$ values to values obtained at day 3–14 in lungs. The $D_q$ values for these experiments were also similar to those obtained at day 3–14 in lungs, and were thus significantly greater than $D_9$ values obtained for line 4T07 in subcutaneous tumours ($P<0.05$). $ID_{90}$ values for line 66/66FAR were also significantly greater than those obtained for line 4T07 ($P<0.05$).

Thus, unlike the situation in early stages of metastasis, there appears to be a difference in sensitivity between line 4T07 and line 66/66FAR in late stage metastases and in subcutaneous tumours. This differences is confirmed by our previously published data indicating that subcutaneous tumours of line 66 are less sensitive to a single moderate dose of melphalan than are line 4T07 tumours (Miller *et al.*, 1991).

### Discussion

We describe here a technique for measuring the drug sensitivity of tumour cells are various stages in the metastastic process, through the use of colony-forming assays to measure the proliferative capacity of tumour cells at various times after treatment. Such techniques are standard for measuring chemocytotoxicity in solid tumours; however, by use of tumour cells carrying drug-resistance markers and thus able to grow in selective medium, thereby preventing growth of the large excess of normal cells, we have been able to apply this technique to cells in occult metastases as well.

Data on the changes in sensitivity of tumour cells in lungs as a function of time after injection need to be seen in relation to the metastatic sequence. In order for tumour cells injected intravenously to form lung colonies, the cells must be arrested and retained in the lung, extravasate, and replicate in lung parenchyma. Previous studies with line 4T07 using similar colony-forming assays have shown that it arrests and grows in lung in a characteristic time-dependent manner which can be related to these several steps of metastasis (Aslakson *et al.*, 1991*a*, 1991*b*). The majority (>80%) of line 4T07 cells injected i.v. lodge very quickly in the lungs (Aslakson *et al.*, 1991*b*), but most of these cells are killed rapidly, so the number of tumour cells drops exponentially for approximately 8 h, with a half time for clearance of 2 to 4 h (Aslakson *et al.*, 1991*a*, 1991*b*). After 8 h the rate of tumour cell loss slows. As suggested by Liotta and DeLisi (1977), this second phase correlates with the extravasation of line 4T07 cells from blood vessels into lung parenchyma (Aslakson *et al.*, 1991*b*). Since melphalan is a drug with a short half-life under physiological conditions (Parsons *et al.*, 1981), the contact of tumour cells with active drug must be of short duration and relatively soon after drug injection. At the time of earliest treatment, 45 min after injection, the tumour cells were nearly all arrested in the lung, but not extravasated. By the time of harvest 24 h after treatment, however, the surviving cells may have completed the extravasation process.

Sometime within 24 h after injection of line 4T07 cells, the number of colony-forming tumour cells starts to rise as cell replication begins to exceed cell death (Aslakson *et al.*, 1991*b*). The number of colony-forming line 4T07 cells increases exponentially between day 1 and at least day 7 after injection: previously measured doubling times in this growth period averaged 21 h (Aslakson *et al.*, 1991*a*, 1991*b*). Thus, when mice were treated with melphalan 3 days after cell injection, the cells were in a period of rapid exponential growth in the lungs.

Grossly visible nodules appear on the surface of lungs of untreated mice approximately 10 to 14 days after i.v. injection of line 4T07. When lungs of mice in experiments depicted in Figure 2, panel d were examined prior to digestion, most lungs contained visible metastases. Although we have not measured the growth rate of colony-forming line 4T07 cells in mice with large tumour burdens in the lung, tumour cell growth rate is likely to slow as tumour nodules become larger. Thus, when mice were treated with melphalan 9 to 15 days after line 4T07 injection, the tumour cells were at this late stage of metastasis.

We have not analysed the time course of growth of untreated line 66FAR cells in the lung. However, studies similar to that described above for line 4T07 have been completed for the closely related line 66c14 (Aslakson *et al.*, 1991*a*).

These studies show a pattern similar to that of line 4T07. Both line 66c14 and 66FAR grow more slowly in the lung that does line 4T07. The doubling time of line 66c14 averages 39 h (Aslakson *et al.*, 1991*b*), whereas line 66FAR grows even more slowly (doubling time of 54 h, not shown). Nevertheless, visible metastatic nodules are found in the lung by day 13 to 20. Thus, when line 66FAR cells were treated with melphalan 3 or 8 days after cell injection, they were in a period of slow expansion of colony-forming cell number in the lungs. By day 14–19, metastatic deposits were large enough to be visible in many mice.

In solid tumours, it is a common observation that a treatment known to cause a relatively large tumour cell kill as determined by colony-forming assay may result in a relatively small effect on tumour growth or host survival time (Steel, 1977). This may be due to a number of factors such as repair of potentially lethal damage *in situ*, rapid regrowth of tumour cells after treatment, and slow clearance of dead or damaged tumour cells *in vivo*. We have previously shown that melphalan treatment of subcutaneous tumours of line 4T07 results in an initial loss of colony-forming cells assayed 24 h after treatment, followed by a gradual increase in colony-forming cells per g tumour in treated mice so as to be indistinguishable from controls by day 14 after treatment (Miller *et al.*, 1991). We describe here that in lung metastases, the difference between the number of colony-forming tumour cells per lung in treated and untreated animals assayed within 1 h after treatment is only slightly more than differences seen on several days after melphalan treatment. This suggests that repair of potentially lethal damage *in situ*, which is likely to take place in the first days after treatment, is of small consequence in this system. However, the percent reduction in the number of metastatic nodules found at the time of necropsy was considerably smaller than the amount of tumour cell kill measured 24–48 h after treatment, indicating the somewhat transient effect of treatment. Although we saw no sign of accelerated regrowth of line 4T07 early after treatment (Figure 1), it is likely that the tumour-burden in treated mice tends to 'catch up' to that in untreated mice as growth slows late in metastasis development. Increased survival time or cure in the ultimately desired effect of treatment, but it is important to determine immediate effects of treatment, even if they are transient, since such information may suggest the type and timing of subsequent effective treatment.

We have shown that tumour cell sensitivity to melphalan varies between cells at different stages in the metastatic process and between cell at early stages of metastasis *vs* cells in palpable subcutaneous tumours. In our experiments, cells treated relatively late in the metastatic process, after nodules had formed, responded very like cells in subcutaneous tumours. Such information obtained for a number of therapeutic agents may lead to combined therapy regimens tailored for specific disease stages.

Some tumour cell lines appear to vary more than others in different stages of metastasis, so that two cell lines may be similar in sensitivity at one stage and differ at another. We have shown that line 66FAR and line 4T07 are very similar in sensitivity to melphalan when cells are treated very soon after i.v. injection and in early stages of growth in lungs, as well as being similarly sensitive *in vitro*. However, in late stages of growth in lungs and in subcutaneous tumours, line 4T07 is more sensitive to melphalan than is line 66FAR, becaue it has a more consistant loss of the low-dose shoulder of the dose-response curve. Indeed, line 4T07 cells in late metastases were actually more sensitive to low doses of drug than were 4T07 cells treated earlier, a result which, although counter-intuitive, is highly reproducible.

As we indicated previously, within the range obtained from replicate experiments, the size of the tumour burden did not systematically affect the dose-response curve parameters describing melphalan sensitivity in either cell line. On the other hand, there were large differences in mean tumour burden between experiments in which mice were treated and sacrificed early (day 0–1), at intermediate times (day 3–4) or late (day 14–15). These differences were particularly pronounced in line 4T07, which grew very rapidly in lungs, even though we injected 10 times as many cells into mice to be sacrificed early *vs* those to be sacrificed late, in order to partly compensate for these differences. The smallest mean tumour burden of $9.9 \times 10^2$ colony-forming tumour cells per lung was determined in an experiment in which $5 \times 10^5$ line 4T07 cells were injected, then harvested 1 day later. The largest mean tumour burden of $2.8 \times 10^5$ colony-forming tumour cells per lung was found in an experiment in which $5 \times 10^4$ line 4T07 cells were injected, then harvested 15 days later. These experiments thus represent a range of tumour burdens of almost 300 fold. However, it does not appear that differences in tumour burden alone can account for the changes in sensitivity to melphalan we have described. Line 4T07 tumour cells harvested in an intermediate time (day 4), from mice having intermediate tumour burdens (averaging $1.6 \times 10^5$ colony-forming cells per lung) were less sensitive to melphalan (larger $D_o$) then those treated early, and also less sensitive (larger $D_q$) than those treated late. Mice bearing line 66FAR had a similar, although somewhat smaller range of tumour burdens (smallest: $7.4 \times 10^3$ colony-forming cells per lung from $5 \times 10^5$ injected, harvested day 1; largest: $4.7 \times 10^5$ from $1 \times 10^5$ injected, harvested day 15). However, even though the tumour burden had become as large at 15 days in line 66FAR as in line 4T07-bearing animals, there was not a similar increase in melphalan sensitivity in those tumour cells.

Further studies will be needed to determine the reasons for these changes in sensitivity at various stages. It is well known that chemotherapeautic agents which are cell-cycle dependent or strongly proliferation dependent are less effective in large tumours when many cells are out of cycle (Steel, 1977). Melphalan is usually described as cell cycle independent; however, it has been shown to be somewhat more cytotoxic to cells in the $M/G_1$ phase in at least one system (Bhuyan *et al.*, 1972). It is more cytotoxic for proliferating cells (Blosmanis *et al.*, 1987). It may be that cell cycle or cell proliferation differences between cells at various stages of metastasis are responsible for some of the differences in sensitivity to melphalan described here. However, the increased sensitivity to melphalan shown by line 4T07 in late stage metastasis and in palpable tumours is unlikely due to *increased* cell proliferation at these stages; in fact, relatively few line 4T07 cells are in cycle in subcutaneous tumours at any given moment (Miller *et al.*, 1987).

Some chemotherapeutic agents may be more or less effective in larger tumours because hypoxic conditions, increased cell contact, or other environmental effects may lead to mechanistically relevant changes in cellular metabolism. Changes in cellular metabolism which may occur at different stage of metastasis could affect sensitivity to melphalan. Alternatively, cells at different metastatic stages may differ in sensitivity to melphalan because of differences in exposure to drug, or differences in ability to repair potentially lethal damage. It is possible that the increased sensitivity of both cell lines to melphalan when treated 45 min after cell injection may be due to increased drug exposure before cells have extravasated into lung parenchyma.

Studies with radiation have indicated that the extent of sublethal damage repair often correlates with the breadth of the shoulder in the acute dose-response curve (Hall, 1988). In the case of melphalan cytotoxicity, Parsons reported that time-dependent repair of certain DNA-cross-links was correlated with resistance to melphalan cytotoxicity at low doses or short exposure times in one of two human melanoma lines (Parsons, 1984). The fact that moderate to large shoulders were seen in dose response curves to melphalan from both of our cell lines at earlier stages of metastasis may suggest that both lines have the ability to repair sublethal damage under these growth conditions. However, with many chemotherapeutic agents, there is little correlation between the extent of the shoulder of a single dose-response curve and the appearance of sublethal damage repair determined by increased survival in split dose experiments (Hall, 1988), suggesting that low dose shoulders reflect other metabolic effects affecting the threshold of drug toxicity such as reduced transport of drug at low concentrations. In the experiments described here, no shoulders or only small shoulders were seen in dose-response curves from four of four late-stage metastasis experiments with line 4T07, and from two of two subcutaneous tumour experiments with line 4T07, but from none of three subcutaneous tumour experiments and three late-stage metastasis experiments with line 66FAR/66. It may be that slow-developing local microenvironmental effects or host reactions to tumour specific to line 4T07 may be instrumental in the loss of these low dose shoulders. Such loss has so far not been seen in a number of *in vitro* culture configurations, suggesting that factors unique to the *in vivo* environment may be involved. Two candidate microenvironmental factors are extent of vascularisation and extent of inflammatory cell infiltration, both of which vary between line 4T07 and line 66FAR/66 tumours. Experiments are in progress to test whether either of these factors contributes to the heightened melphalan sensitivity.

We thank Mr Martin Lehotan, Ms Jean Bukowski, and Ms Jill Werdell for technical assistance, and Ms Margaret Peterson and Ms Janita Brown for manuscript preparation. This work was supported by the United States Public Health Service grant CA 27419.

### References

ASLAKSON, C.J., RAK, J.W., MILLER, B.E. & MILLER, F.R. (1991*a*). Differential influence of organ site on three subpopulations of a single mouse mammary tumor at two distinct steps in metastasis. *Int. J. Cancer*, **47**, 466–472.

ASLAKSON, C.J., MCEACHERN, D., CONAWAY, D.H. & MILLER, F.R. (1991*b*). Inhibition of lung colonization at two different steps in the metastatic sequence. *Clin. Expl. Metastasis*, **9**, 139–150.

BHUYAN, K., SCHEIDT, L.G. & FRASER, T.J. (1972). Cell cycle phase specificity of antitumor agents. *Cancer Res.*, **32**, 398–407.

BLOSMANIS, R., WRIGHT, J.A. & GOLDENBERG, G.J. (1987). Sensitivity to melphalan as a function of transport activity and proliferative rate in BALB/c fibroblasts. *Cancer Res.*, **47**, 1273–1277.

DEXTER, D.L., KOWALSKI, H.M., BLAZAR, B.A., FLIEGEL, Z., VOGEL, R. & HEPPNER, G.H. (1978). Heterogeneity of tumor cells from a single mouse mammary tumor. *Cancer Res.*, **38**, 3174–3181.

FIDLER, I.J. (1978). Tumor heterogeneity and the biology of cancer invasion and metastasis. *Cancer Res.*, **38**, 2651–2660.

FREI, E., TEICHER, B.A., HOLDEN, S.A., CATHCART, N.S. & WANG, Y. (1988). Preclinical studies and clinical correlation of the effect of alkylating dose. *Cancer Res.*, **48**, 6417–6423.

HALL, E.J. (1988). *Radiobiology for the Radiologist*. J.B. Lippencott Co.: Philadelphia.

HEPPNER, G.H., DEXTER, D.L., DENUCCI, T., MILLER, F.R. & CALABRESI, P. (1978). Heterogeneity in drug sensitivity among tumor cell subpopulations of a single mammary tumor. *Cancer Res.*, **38**, 3758–3763.

LIOTTA, L.A. & DELISI, C. (1977). Method for quantitating tumor cell removal and tumor cell-invasive capacity in experimental metastases. *Cancer Res.*, **37**. 4003–4008.

MILLER, B.E., MILLER, F.R., WILBURN, D. & HEPPNER, G.H. (1987). Analysis of tumour-cell composition of tumours composed of paired mixtures of mammary tumour cell lines. *Br. J. Cancer*, **56**, 561–569.

MILLER, B.E., MILLER, F.R. & HEPPNER, G.H. (1989*a*). Heterogeneity of tumor cell sensitivities: implications for tumor response. In *Prediction of Tumor Treatment Response*. Chapman, J.D., Peters, L.J. & Withers, H.H. (eds), p. 227–238. Pergamon Press: New York.

MILLER, B.E., MILLER, F.R. & HEPPNER, G.H. (1989*b*). Therapeutic pertubation of the tumor ecosystem in reconstructed heterogeneous mouse mammary tumors. *Cancer Res.*, **49**, 3747–3753.

MILLER, B.E., ASLAKSON, C.J. & MILLER, F.R. (1990). Efficient recovery of clonogenic stem cells from solid tumors and occult metastatic deposits. *Invasion Metastasis*, **10**, 101–112.

MILLER, B.E., MACHEMER, T., LEHOTAN, M. & HEPPNER, G.H. (1991). Tumor subpopulation interactions affecting melphalan sensitivity in palpable mouse mammary tumors. *Cancer Res.*, **51**, 4378–4387.

MILLER, F.R., MILLER, B.E. & HEPPNER, G.H. (1983). Characterization of metastatic heterogeneity among subpopulations of a single mouse mammary tumor: heterogeneity in phenotypic stability. *Invasion Metastasis*, **3**, 22–31.

PARSONS, P.G. (1984). Dependence on treatment time on melphalan resistance and DNA cross-linking in human melanoma cell lines. *Cancer Res.*, **44**, 2773–2778.

PARSONS, P.G., CARTER, F.B., MORRISON, L. & SR. REGIUS MARY. (1981). Mechanism of melphalan resistance developed *in vitro* in human melanoma cells. *Cancer Res.*, **41**, 1525–1534.

SCHMID, F.A., OTTER, G.M. & STOCK, C.C. (1980). Resistance patterns of Walker carcinosarcoma 256 and other rodent tumors to cyclophosphamide and l-phenylalanine mustard. *Cancer Res.*, **40**, 830–833.

STEEL, G.G. (1977). *Growth Kinetics of Tumours*. Clarendon Press: Oxford.